# Surgical Treatment Outcomes of Unresolved Osgood-Schlatter Disease in Adolescent Athletes

**DOI:** 10.1155/2021/6677333

**Published:** 2021-03-17

**Authors:** Frederick Mun, William L. Hennrikus

**Affiliations:** ^1^Penn State College of Medicine, Hershey, PA 17033, USA; ^2^Penn State Hershey Medical Center, Bone and Joint Institute, Hershey, Pennsylvania 17033, USA

## Abstract

The purpose of this case series is to report the outcomes of ossicle excision and tubercleplasty for unresolved Osgood-Schlatter disease that has failed conservative treatment in six adolescent athletes. A retrospective chart review was completed, and data collected include age at onset of symptoms, age at surgery, sex, laterality, mechanism of injury, conservative treatment regimen, radiographic findings, sports played, time to return to sport, length of follow-up, and Lysholm score. Surgery involved an open ossicle excision, tubercleplasty, and repair of the patellar tendon to bone using a suture anchor. Postoperatively, patients were allowed to fully weight-bear in an extension knee brace for 4 weeks and then allowed to gradually resume activity. Four males and 2 females were studied. The right knee was involved in 3 cases and the left knee in 3. The average age at onset of symptoms was 15.8 (range 12-18) and at surgery was 17.3 (range 17-18). Radiographic findings included a large bump in 4 cases, an ossicle in 2, and free fragments at the tendon insertion in 3. Sports played included basketball, football, running, and dancing. All patients returned to sports at an average of 21 weeks and 6 days postsurgery (range 8-56). The average length of follow-up was 14.2 weeks (range 5-27). The average Lysholm score postsurgery was 97.2 (range 94-100). Surgical treatment of unresolved Osgood-Schlatter disease was successful in all patients. No patients reported any postoperative complications or additional surgery. For skeletally mature and symptomatic patients, we recommend removal of the ossicle and adjacent bursae, smoothing the bump, and repairing the patellar tendon to bone.

## 1. Introduction

Osgood-Schlatter disease is a traction apophysitis of the tibial tubercle caused by repetitive mechanical strain by the quadriceps femoris muscle. Painful inflammation develops at the patellar ligament insertion point on the tibial tubercle [1, 2]. Repetitive strain may cause avulsion fractures, eventually leading to sclerosis and fragmentation of the tibial tubercle. Rising and expansion of the tibial tubercle and soft-tissue swelling may produce persistent pain [3]. Osgood-Schlatter disease is often a self-limiting condition that is treated conservatively and affects children and adolescent athletes during growth spurts between the ages of 9 and 15 [1]. Conservative therapy, such as ice, immobilization, and rest, is the preferred method of treatment. Nonsteroidal anti-inflammatory drugs may be used for acute symptom relief [1, 2, 4].

After skeletal maturity, complications may arise and a few patients can still have severe and persisting pain despite conservative therapy [3, 5, 6]. In such cases, we hypothesized that surgical intervention could potentially help to alleviate symptoms. Previous studies have shown that open, arthroscopic, or direct bursoscopic excision of the painful tibial tubercle bump can be used [6–10]. Surgical treatment outcomes for unresolved, painful Osgood-Schlatter disease in adolescent athletes have only been reported by several authors [6, 11]. The aim of this study is to report the outcomes of surgical treatment by open ossicle excision and tubercleplasty in persistent Osgood-Schlatter disease in teenage athletes.

## 2. Case Presentation

### 2.1. Methods

The Institutional Review Board at the College of Medicine approved the study. Six heterogeneous patients, from January 1, 2008, to January 1, 2018, who underwent ossicle excision and tibial tubercleplasty for unresolved Osgood-Schlatter disease, were included in our study. Various patient characteristics were collected and analyzed including age at onset of symptoms, age at surgery, sex, sports played, laterality, mechanism of injury, conservative treatment regimen, radiographic findings, length of follow-up, Lysholm knee score, time until return to sports, and complications. Descriptive statistics, including frequency, mean, minimum, and maximum values, were calculated for the patient's age, mechanism of injury, length of follow-up, Lysholm knee score, and time until return to sports. Frequency was determined for the patient's sex, sport, laterality, conservative treatment regimen, and radiographic findings.

Surgery was performed by a fellowship-trained pediatric orthopaedic surgeon who has >30 years of experience. The surgical procedure included an open ossicle excision, tubercleplasty, and repair of the patellar tendon to bone using a suture anchor in each case. A direct anterior incision was avoided to minimize postoperative pain with kneeling. A longitudinal incision was made over the lateral aspect of the patellar tendon. The patellar tendon was then reflected medially to gain access to the ossicle and bump. Postoperative treatment allowed for full weight-bearing in an extension knee brace for 4 weeks. Nonsteroidal anti-inflammatory drugs were used for acute symptom relief. Patients were allowed to gradually return to normal activity and sport.

### 2.2. Results

**Figure 1:**
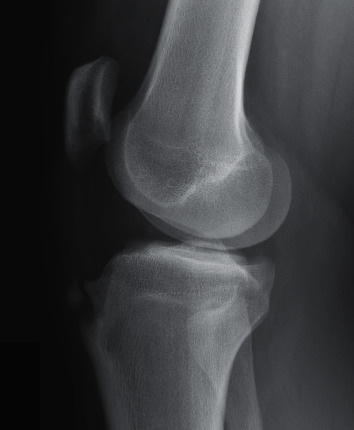
Preoperative, 17-year-old male with bony fragmentation and bump due to Osgood-Schlatter disease on the right knee.

**Figure 2:**
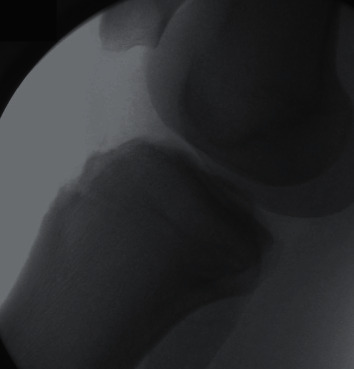
Postoperative, 17-year-old male with resolved Osgood-Schlatter disease on the right knee.

Four males and 2 females, with closed growth plates, were studied. The right knee was involved in 3 cases, and the left knee in 3. The average age at onset of symptoms was 15.8 (range 12 to 18). The average age at surgery was 17.3 (range 17 to 18). Five patients presented with chronic pain worsening over an average of 3.4 years (range 1-5 years). All 5 were initially treated conservatively, with rest, ice, and nonsteroidal anti-inflammatory drugs as needed. One patient presented after a direct blow to the knee during a football game and had no history of chronic knee pain. Radiographic findings included a large bump in 4 cases, an ossicle in 2 cases, and free fragments at the tendon insertion in 3 cases. Sports played included basketball, football, running, and dancing. All 6 patients were high school varsity athletes and successfully returned to their sport at full capacity at an average of 21 weeks and 6 days postsurgery (range 8 weeks and 6 days to 56 weeks). The average length of follow-up was 14.2 weeks (range 5 weeks to 27 weeks). The average postsurgery Lysholm score in September 2020 was 97.2 (range 94 to 100).  Table 1 shows patient demographics and characteristics. Figures 1 and 2 show pre- and postoperative radiographic findings of one of the patients, exhibiting the successful removal of bony fragmentation and residual bump.

## 3. Discussion

Osgood-Schlatter disease classically presents with a history of activity-related trauma that causes pain over the tibial tubercle and patellar tendon. On physical exam, there is a bump, with tenderness and swelling over the tibial tubercle. Pain can often be reproduced by extension of the leg or kneeling [1, 2]. The pain is relieved with rest and exacerbated with activity. It is often associated with athletes who play sports like volleyball, basketball, gymnastics, and soccer which require repetitive flexion and extension of the knee [1, 2, 12]. In fact, Kujala et al. reported that among 193 adolescent athletes, 41 (21.2%) were diagnosed with Osgood-Schlatter disease, while among 196 sedentary adolescents, only 9 (4.5%) were diagnosed [12]. Males will typically become symptomatic between ages 12 and 15, while females will between at ages 8 and 12 [10]. The most commonly reported rate of prevalence of Osgood-Schlatter disease is 9.8% (11.4% in males and 8.3% in females) [4]. However, there are also recent studies that have demonstrated the gap in prevalence is closing due to increasing numbers of female adolescent athletes [4, 13].

Symptoms can persist for 12 to 18 months and commonly resolve with skeletal maturity [1]. Conservative treatments include controlled immobilization, stretching, and reduction of activity, which are the preferred methods of treatment. Anti-inflammatory drugs can also be used for acute symptom relief [1, 2]. There have been several studies on injection of dextrose, lidocaine, or autologous-conditioned plasma into the patellar tendon to provide symptomatic relief as well [14–16]. Although conservative therapy is the mainstay treatment of Osgood-Schlatter disease, surgical intervention may be necessary for patients whose symptoms do not resolve with skeletal maturity [3, 6]. A small percentage of patients develop an ossicle and painful bump despite closure of the growth plates and are indicated for surgery [6–8]. At our institution, 6 patients presented with persistent symptoms past skeletal maturity and were indicated for surgery.

Approximately 5% of patients will develop unresolving, painful Osgood-Schlatter disease and may require surgery [5, 8, 17]. The literature has cited many different surgical procedures for treatment including open procedures (such as drilling of the tibial tubercle, excision of the tibial tubercle, longitudinal incision in the patellar tendon, tibial sequestrectomy, and bone peg insertion), arthroscopic procedures, and bursoscopic procedures [17].

Bosworth et al. discussed efficacy of bone peg in the tubercle to expedite fusion. Although this resolved symptoms in most cases, the bumpy prominence still persisted postoperatively [18].

Thomson first reported success with utilizing a longitudinal incision in the patellar tendon to excise the bony prominence [19]. Flowers and Bhadreshwar reported that postoperatively 95% of their patient cohort had pain relief and 86% had a reduced bump [8]. Orava et al. reported that 80% of their cohort who underwent either removal of ossicle or excision/drilling of the tibial tubercle had excellent or good results [20]. Glynn et al. discussed the efficacy of excision of the prominent tubercle, as opposed to drilling of the tibial tubercle [7]. Some authors have also explored arthroscopic and bursoscopic methods as viable alternatives to open procedures for Osgood-Schlatter disease, due to scar tissue and the potential damage to the patellar tendon [9, 21].

In 2007, Weiss et al. reported that of the 15 patients who underwent ossicle resection with tibial tubercleplasty, 12 (80%) successfully recovered and returned to sports. We followed the protocol discussed by Weiss et al. [6]. Our institution utilized open ossicle excision, tubercleplasty, and repair of the patellar tendon to bone using a suture anchor. All 6 patients successfully returned to their sport at full capacity postsurgery. The average Lysholm score in September 2020 was 97.2 (range 94 to 100). Surgical treatment may successfully allow adolescent athletes to return to activity. No patients reported postoperative pain with kneeling, a limp, need for a cane, locking sensations, giving way sensations, pain, swelling, difficulty climbing stairs, or difficulty squatting. Furthermore, there were no postoperative complications, such as infections. No patients required additional surgery.

Limitations associated with this study include its retrospective design, small sample size, and absence of a comparative group. Future studies may overcome these limitations by lengthening the study's time period or by conducting multicenter studies to potentially increase the number of included patients.

## 4. Conclusion

Based on the study findings, we recommend conservative treatment for adolescent athletes when they initially present with symptoms of Osgood-Schlatter disease. However, if symptoms persist at skeletal maturity, surgical intervention (via an open ossicle excision, tubercleplasty, and repair of the patellar tendon to bone using a suture anchor) is an effective option to resolve symptoms and return the athlete to sports.

## Figures and Tables

**Table 1:** Demographics of patients who underwent surgery for unresolved Osgood-Schlatter disease.

Patient	Age at surgery	Sex	Sport	Side	Mechanism of injury	Conservative treatment	Preoperative radiograph	Postoperative radiograph	Length of follow-up	Lysholm knee score	Returned to sport?	Time until sport return
1	17	F	Dance	Right	Chronic pain worsening for 3 years	Rest, ice, ibuprofen PRN	Residual bump	Resolved OSD	6 weeks and 5 days	96	Yes	14 weeks and 5 days
2	17	F	Dance	Left	Chronic pain worsening for 1 year	Rest, ice, ibuprofen PRN	Ossicle+free fragments	Resolved OSD	27 weeks and 1 day	95	Yes	56 weeks
3	17	M	Track	Right	Chronic pain worsening for 4 years	Rest, ice, ibuprofen PRN	Bump+free fragments	Resolved OSD	14 weeks and 5 days	94	Yes	14 weeks and 5 days
4	17	M	Basketball+football	Right	Chronic pain worsening for 4 years	Rest, ice, ibuprofen PRN	Residual bump	Resolved OSD	8 weeks and 6 days	99	Yes	8 weeks and 6 days
5	18	M	Basketball	Left	Chronic pain worsening for 5 years	Rest, ice, ibuprofen PRN	Residual bump	Resolved OSD	26 weeks and 6 days	99	Yes	26 weeks and 6 days
6	18	M	Football	Left	Direct blow to the knee during football game	N/A	Ossicle+free fragments	Resolved OSD	5 weeks	100	Yes	10 weeks

## Data Availability

The data used to support the findings of this study are restricted in order to protect patient privacy.

## Supplementary Materials

Supplementary Materials We have included the recommended Joanna Briggs Institute's checklist for our case series.
