# Supplementary material for: Surgical Treatment Outcomes of Unresolved Osgood-Schlatter Disease in Adolescent Athletes
Source: Case Rep Orthop. 2021 Mar 17;2021:6677333. doi: 10.1155/2021/6677333 (PMC7990524; doi:10.1155/2021/6677333)
Supplement: Supplementary Materials — We have included the recommended Joanna Briggs Institute's checklist for our case series. [file 6677333.f1.docx]

JBI Critical Appraisal Checklist for Case Series

1. Were there clear criteria for inclusion in the case series? Yes
2. Was the condition measured in a standard, reliable way for all participants included in the case series? Yes
3. Were valid methods used for identification of the condition for all participants included in the case series? Yes
4. Did the case series have consecutive inclusion of participants? Yes
5. Did the case series have complete inclusion of participants? Yes
6. Was there clear reporting of the demographics of the participants in the study? Yes
7. Was there clear reporting of clinical information of the participants? Yes
8. Were the outcomes or follow up results of cases clearly reported? Yes
9. Was there clear reporting of the presenting site(s)/clinic(s) demographic information? Yes
10. Was statistical analysis appropriate? Yes
